# Physical therapy in occupational health and ergonomics: practical applications and innovative research approaches

**DOI:** 10.1590/bjpt-rbf.2014.0193

**Published:** 2016-11-16

**Authors:** Rosimeire S. Padula, Ana B. Oliveira, Rodrigo L. Carregaro, Tatiana O. Sato

**Affiliations:** 1 Masters and Doctoral Programs in Physical Therapy, Universidade Cidade de São Paulo (UNICID), São Paulo, Brazil; 2 Department of Physical Therapy, Universidade Federal de São Carlos (UFSCar), São Carlos, SP, Brazil; 3 Rehabilitation Sciences Graduate Program, School of Physical Therapy, Universidade de Brasília (UnB), Campus UnB Ceilândia, Brasilia, DF, Brazil

**Keywords:** intervention study, workplace, physical therapy, public health, evidence based practice

This letter to the editor aims to enlighten the readers of the *Brazilian Journal of Physical Therapy* (BJPT) on the relevance and conceptual framework, practical applications, current scenario, and advances of physical therapy in occupational health and ergonomics. Additionally, this letter presents challenges to be overcome and perspectives for physical therapists as well as the scientific community.

We have witnessed the progress and achievements of the BJPT over the last two decades^1^. These advances have been supported by the researchers’ expertise, the quality of publications, and the scientific evolution of the physical therapy profession worldwide.

It is well known that the physical therapy profession has a diversity of knowledge related to its specialties and public health demands. This diversity can be attributed to aspects such as relevant clinical outcomes, target populations, and a wide variety of settings in which physical therapists can work^2^.

In terms of historical context, physical therapy has been influenced by economic, cultural, and educational aspects^3^ of public and research policies that prioritize some fields of expertise over others. Among physical therapy’s specialties, occupational health and ergonomics is a novel example which is both increasing in clinical relevance and dissemination by professionals in a range of settings. This relevance can be easily seen by the increasing number of physical therapists involved in occupational health and ergonomics initiatives worldwide.

The impact of this relevance has also been reflected by an increase in demand for improvements in worker health and company productivity. It is important to point out that advanced skills and clinical competencies are necessary to improve our professional capabilities and to expand the presence of physical therapists in different occupational settings. However, our professional practice is still guided by weak scientific evidence regarding the effectiveness of intervention strategies and the validity of instruments used for the identification of workplace risk factors, as well as for the evaluation of physical and functional worker health. Why is this so? Some reasons are presented below in order to understand this scenario.

Contrasting with the broad spectrum of professional activities promoted by physical therapists in occupational health and ergonomics, scientific research is only at an incipient stage. Although research within occupational health and ergonomics has been conducted worldwide, there are few physical therapists in this field, which has resulted in a limited number of published studies compared with other specialties such as musculoskeletal and cardiorespiratory physical therapy.

The World Confederation for Physical Therapy (WCPT) supported the creation of a physical therapy network^4^ composed of an international research group to integrate physical therapists who are working in occupational health and ergonomics. The initial goal of this network was to engender discussion among professionals and to organize a meeting during the World Physical Therapy Congress. We can thus point out that physical therapy in occupational health and ergonomics in Brazil and worldwide share similar aspects and need to expand and solidify.

In Brazil, the duties of physical therapists working in occupational health and ergonomics were approved by COFFITO Resolution no. 403 in 2011^5^, which provided clarity on the roles of physical therapists within this specialty. This Resolution^5^ ^,^ ^6^, which is similar to those used in other countries such as Australia, The Netherlands, Canada, and the United States, sets out the skills and competencies required for physical therapists to implement health education programs, physical capacity and functional assessments, job and workplace analysis, training of motor abilities, risk control, and physical exercise programs^6^ ^,^ ^7^. Resolutions such as these demonstrate the importance of this specialty internationally.

Although recognized and represented by a large number of physical therapists in the workplace, the specialty is still small in terms of number of publications. The lack of research conducted by physical therapists in occupational health and ergonomics confirms the low representation compared to more consolidated fields in the scientific context. This scenario has a direct impact on the scientific output in the field of occupational health and ergonomics^2^ ^,^ ^8^. As an example, we can emphasize the small number of clinical trials registered in the PEDro database, which includes less than 600 publications on occupation health and ergonomics (search conducted in March 2016), making it the smallest sub-discipline within all fields of physical therapy. Nevertheless, this does not mean that occupational health and ergonomics in physical therapy does not have an impact on scientific research. A fundamental question is related to the characteristics of this field of research, which focuses on studies analyzing determinants of work with cross-sectional and longitudinal designs using epidemiological and biomechanical approaches.

A recent discussion paper presented the challenges faced by researchers from occupational health and ergonomics attempting to conduct randomized controlled trials (RCT) as this research design is the “gold standard” for evidence-based practice^9^ in the workplace. The main challenges are related to organizations, many of which have concerns about RCT research design, such as: 1) the organization objects to randomly allocating their employees to an intervention or control group; 2) the organization wants to target all employees with an intervention; 3) the organization wants to adjust the intervention protocol; and 4) the organization is subject to internal or external changes. The consequences of these challenges may produce confounding results due to unreliable randomization, selection bias, or lack of a control group. Consequently, to overcome these concerns, certain study designs are preferable, such as stepped-wedge, propensity scores, instrumental variables, multiple baseline design, interrupted time series, difference-in-difference, and regression discontinuity^9^. These suboptimal designs are much more likely to be biased compared with high quality, randomized controlled trials, but given the impossibility of conducting clinical trials, these research designs can be very useful.

Therefore, this letter calls upon professionals and researchers to strengthen the relevance and quality of research within this field. Additionally, it seeks to make editors and researchers aware of the importance of high quality studies for physical therapy in occupational health and ergonomics and of other study designs for the advancement of scientific knowledge. This awareness is corroborated by current health demands such as aging of the population, functional assessment of workers, increased participation of women in the workplace and its impact on work design and daily life activities, human functionality and the influence of workplace risk factors, the growth of chronic diseases, and the importance of the discussion of the physical therapist’s role in prevention, health, and well-being programs. Collaboration between professionals and researchers is crucial to redirect and change the national scenario in the long term.

Our intent with this letter has been to focus on the demands and scientific progress of physical therapy in occupational health and ergonomics and to raise awareness in the academic community concerning the role of the physical therapist and of research within this field. Moreover, we intend to encourage the development of further studies with different designs and highlight the applicability and potential of each model for evidence-based physical therapy and the interface with occupational health and ergonomics.

We believe that our involvement and experience is innovative, therefore we invite our research fellows and colleagues to contribute to this field in order to help the physical therapy profession to broaden the body of knowledge and increase its research productivity and impact, taking into account the peculiarities and characteristics of each field of research.
